# Chromatin accessibility landscapes revealed the subgenome-divergent regulation networks during wheat grain development

**DOI:** 10.1007/s42994-023-00095-8

**Published:** 2023-02-10

**Authors:** Hongcui Pei, Yushan Li, Yanhong Liu, Pan Liu, Jialin Zhang, Xueni Ren, Zefu Lu

**Affiliations:** Institute of Crop Sciences, Chinese Academy of Agricultural Sciences, Beijing, 100081 China

**Keywords:** Wheat, Chromatin accessibility, Subgenome-divergence, Regulatory network, Grain development

## Abstract

Development of wheat (*Triticum aestivum* L*.*) grain mainly depends on the processes of starch synthesis and storage protein accumulation, which are critical for grain yield and quality. However, the regulatory network underlying the transcriptional and physiological changes of grain development is still not clear. Here, we combined ATAC-seq and RNA-seq to discover the chromatin accessibility and gene expression dynamics during these processes. We found that the chromatin accessibility changes are tightly associated with differential transcriptomic expressions, and the proportion of distal ACRs was increased gradually during grain development. Specific transcription factor (TF) binding sites were enriched at different stages and were diversified among the 3 subgenomes. We further predicted the potential interactions between key TFs and genes related with starch and storage protein biosynthesis and found different copies of some key TFs played diversified roles. Overall, our findings have provided numerous resources and illustrated the regulatory network during wheat grain development, which would shed light on the improvement of wheat yields and qualities.

**Supplementary Information:**

The online version contains supplementary material available at 10.1007/s42994-023-00095-8.

## Introduction

Wheat (*Triticum aestivum* L.) is one of the most important staple crops for global food security, providing more than a fifth of the calories and protein consumed by humans (http://faostat.fao.org). Wheat grains are mainly composed of pericarp, endosperm and embryo, of which the endosperm is the main source of white flour. The endosperm is a storage organ that accumulates a large quantity of starch and proteins during grain development (Gu et al. 2015; Liu et al. 2018; She et al. 2010). Therefore, elucidation of the transcriptional regulatory networks during wheat grain development is important to understand the molecular basis of wheat yield and quality and discover novel key regulatory factors.

Starch and protein are the main nutrient components of wheat grain. A variety of starch biosynthesis genes have been characterized, including ADP-glucose pyrophosphorylases (AGPases), ADP-glucose (ADPG) transporter, granule-bound starch synthases (GBSSs), starch synthases (SSs), Starch branching enzymes (SBEs), debranching enzymes (DBEs), starch/α-glucan phosphorylases (PHOs), disproportionating enzymes (DPEs), and protein targeting to starch (PTST), which coordinately work in starch biosynthesis during wheat grain development (Huang et al. 2021; Kang et al. 2013; Wang et al. 2019; Yamamori et al. 2000). Starch is synthesized by plants using the enzyme AGPases that reacts glucose 1-phosphate with ATP to form ADPG, the ADPG were subsequent synthetized to amylose and amylopectin with different enzymes. Most of the storage proteins in grains are gluten, including high molecular weight glutenin subunits (HMW-GSs), low molecular weight glutenin subunits (LMW-GSs) and gliadins, which influence the dough property (Liu et al. 2005; Ren et al. 2022). The expressions of those key genes in starch and/or gluten biosynthesis pathways are tightly related with grain yield and quality. A group of transcriptional factors, such as TaRSR1 and TabZIP28, were found to be involved in regulating the expression of genes related to starch synthesis in wheat (Liu et al. 2016; Song et al. 2020), and TaGAMyb, TaFUSCA3 and PBF-D regulate different HMW-GS gene (*Glu-1Dy*, *Glu-1Bx7* and *Glu*-*1*) expression through binding specific DNA motifs (Guo et al. 2015; Sun et al. 2017; Zhu et al. 2018). Besides, many TFs, such as TaNAC019 control glutenin and starch accumulation by targeting both HMW-GS genes and the starch synthase gene *TaSSIIa* to synergetic regulation of wheat yield and quality (Gao et al. 2021; Liu et al. 2020).

Wheat grain development can be typically defined into three stages: the pregrain-filling phase for 10 days after pollination, grain-filling phase for 10–20 days after pollination and desiccation phase then follows beyond 30 days after pollination (Wan et al. 2008). The transition of different grain development phase is accompanied by dramatic transcriptional and physiological changes. However, little is known about their regulatory mechanisms and contribution of each subgenome in allohexaploid wheat. Accessible chromatin regions are putative *cis*-regulatory elements (CREs) that are targeted by TFs and play vital roles in transcriptional regulation. It is reported that the CRE variants are associated with many important agronomic traits (Adamski et al. 2021; Deplancke et al. 2016; Kouzarides 2007; Liu et al. 2021; Lu et al. 2017, 2019; Rodgers-Melnick et al. 2016; Tian et al. 2021; Wang et al. 2021b). In this study, we unraveled the regulatory network underlying wheat grain development by combing ATAC-seq (Assay for Transposase Accessible Chromatin sequencing) and RNA-seq with samples from a series grain developmental stages. Our studies have not only revealed the chromatin accessibility landscapes of wheat grain development, but also provided valuable insights into the dynamic regulatory network mediated by important transcription factors and the divergences of the three subgenomes during grain development in the allohexaploid wheat.

## Results

### Chromatin accessibility changes are associated with differential transcriptomic expressions during grain development

**Fig. 1:**
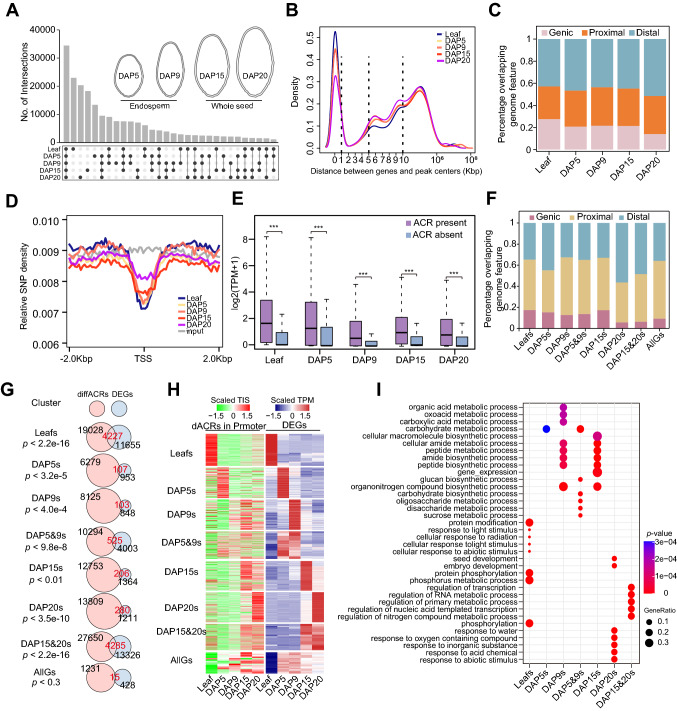
Chromatin accessibilities are relative to the differential gene expression during wheat grain development. **A** UpSet Plot showing the ACR distribution across five tissues (DAP, days after pollination). **B** Density plot showing the distribution between ACR centers and their nearest genes. Black dot line indicates 10 Kb away from genes. **C** Distribution of ACRs on different genomic features. Genic, ACRs with their centers in gene bodies; Proximal, ACRs with their centers <  = 2 Kb upstream of TSS or <  = 2 Kb downstream of TES; Distal, ACR with their centers > 2 Kb away from genes. **D** Distribution of sequence variations around ACR centers. SNPs from Zhou et al*.* were used. **E** The expression of genes with ACRs in their 2 Kb nearby regions (ACR present) and without ACRs in their 2 Kb nearby regions (ACR absent) across the five tissues. **F** Distribution of eight differential ACR (diffACR) clusters on different genomic features. The definition of each group is same with **C**. **G** The overlapping information of eight diffACR clusters related genes and eight DEG clusters. **H** Chromatin accessibilities of regions from 2 Kb upstream to 100 bp downstream of TSS and associated genes’ expression levels. Chromatin accessibilities were represented by Tn5 transposome integration sites (*TISs*) and expression levels by transcripts per million (*TPM*). **I** Gene ontology (*GO*) enrichment analysis for the overlapped gene groups in **G**

Wheat grains are mainly composed of endosperm and embryo, we performed ATAC-seq and RNA-seq with endosperm or seeds in 5, 9, 15, and 20 days after pollination (DAP) to explore the chromatin accessibility and transcriptomic expression dynamics during wheat grain development, and used leaves as control. A total of 103,516, 108,846, 101,620, 105,578 and 104,407 high confident accessible chromatin regions (ACRs) were identified in leaf and grain in DAP5, DAP9, DAP15 and DAP20, respectively (Fig. 1A). All the ACRs from the five tissues are mostly enriched around the TSS regions (Supplementary Fig. 1A) and showed high correlation coefficient (> 0.80) between two biological replicates (Supplementary Fig. 1B–D). Similar with the ACR distribution in other large genome species, a significant faction of ACRs distributed 10 Kb away from genes apart from most of ACRs located near the gene (Fig. 1B). We then grouped the ACRs as genic ACRs (genic ACRs, overlapping with genebody), proximal ACRs and distal ACRs according the distance between ACR centers and their nearest genes. For definition of proximal ACRs and distal ACRs, we employed different distance cutoffs (2 Kb, 4 Kb, 5 Kb, 6 Kb, 8 Kb, 10 Kb), and those within each cutoff away from genes as proximal ACRs, and those larger than cutoff away from genes as distal ACRs (Supplementary Fig. 2). Interestingly, the proportion of distal ACRs in grain samples were all much higher than in leaf except in DAP15 with 6 Kb as the cutoff (Supplementary Fig. 2). When using 2 Kb to distinguish them, more than 50% of ACRs were predominately enriched in the distal regions of genes in DAP20 (Fig. 1C). These results suggested that during grain development, more distal regions were predominantly opened and these may be critical for the expression of genes regulating starch and storage protein biosynthesis.

We further examined the sequence variations around ACRs using the reported wheat whole-genome genetic variation map (VMap 1.0) (Zhou et al. 2020). To avoid the influence of biased signals around genes, we only picked up ACR whose centers are > 2 Kb away from genes. The results showed that lower sequence variation around ACR centers (Fig. 1D), which suggested those ACRs are functionally important. Interestingly, we found that variations were higher in DAP20 than the earlier stages, indicating a lower selection pressure of those genes.

The chromatin accessibility changes are usually associated with differential transcriptomic expressions (Chen et al. 2019; Pajoro et al. 2014). To study the relationship between chromatin accessibilities and gene expressions, we classified the total wheat genes based on the presences of ACRs within 2 Kb away from genes. The results showed that the expression of genes associated with ACRs were significantly higher than those without ACRs in all five tissues (Fig. 1E). To identify the putative CREs responsible for the transcriptomic expression changes, we identified the differential ACRs (diffACRs) by pair-wise comparisons and then grouped the diffACRs into eight specific clusters, including leaf-specific diffACRs (Leafs), DAP5 specific (DAP5s), DAP9 specific (DAP9s), DAP5 and DAP9 specific (DAP5&9s), DAP15 specific (DAP15s), DAP20 specific (DAP20s), DAP15 and DAP20 specifics (DAP15&20s) and all grain specific (AllGs). In total, we identified 31,000, 8346, 9754, 13,829,15,974, 17,253, 39,725 and 1287 specific diffACRs in leafs, DAP5s, DAP9s, DAP5&9s, DAP15s, DAP20s, DAP15&20s and AllGs groups, respectively (Supplementary Fig. 3A and Supplemental Data Set1). The diffACRs were more enriched in the distal regions than the total ACRs, indicating accessibilities of distal regions were more volatile during grain development (Fig. 1F). We further divided DEGs into eight clusters using the similar strategy and identified the overlaps between DEGs and diffACRs associated genes, which revealed higher overlapping rates compared to the random shuffled gene sets in all groups except AllGs (Fig. 1G, Supplementary Fig. 3B, and Supplemental Data Set2). The chromatin accessibilities of promoter regions (– 2 Kb upstream to 100 bp downstream of TSS sites) and the expression of each overlapped gene cluster showed similar trends (Fig. 1H), which further confirmed that the chromatin accessibility changes are associated with differential transcriptomic expressions during wheat grain development.

To clarify the biological processes mediated by each gene group, we performed gene ontology (GO) enrichment analysis. The results showed that leaf-specific genes were highly enriched in phosphorylation and response to light or abiotic stimulus, while the DAP5, DAP9 and DAP5&9 specific genes were highly enriched in carbohydrate, sucrose metabolic process, in addition, amide biosynthesis and nitrogen compound metabolic process regulation were enriched in the overlapped genes specific in DAP15 and/or DAP20 (Fig. 1I). These enriched GO terms were consistent with the grain development processes.

### Subgenome-divergent regulation during wheat grain development

**Fig. 2:**
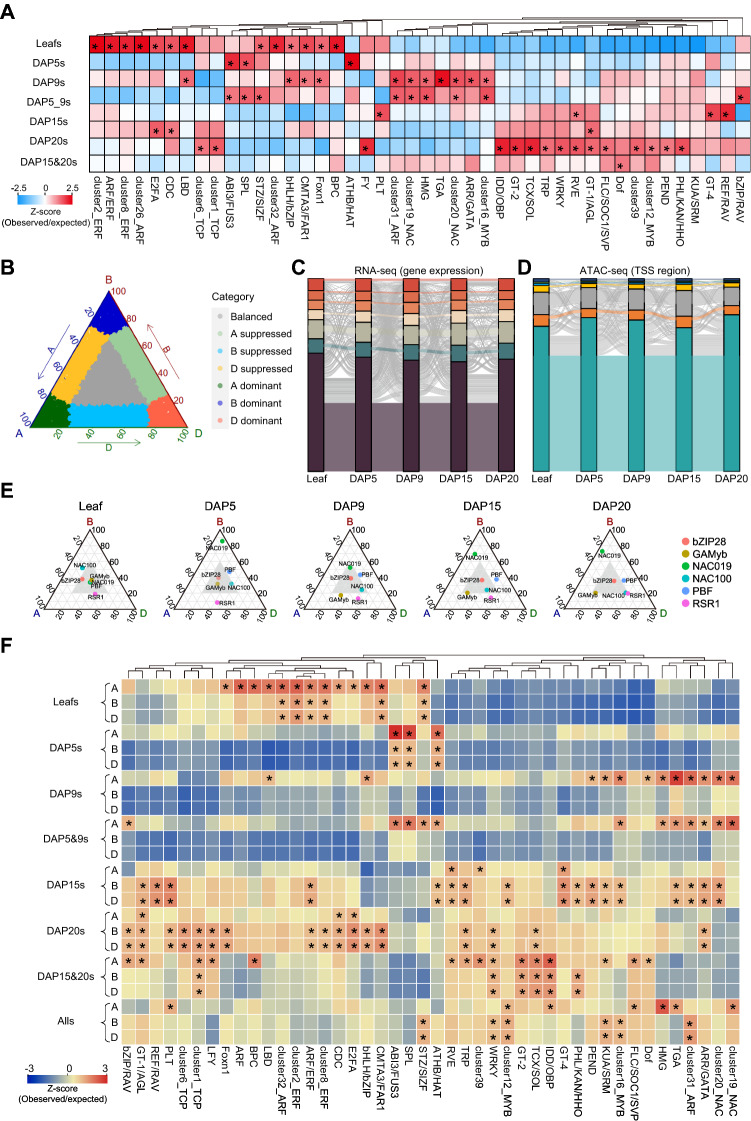
Subgenome-divergent regulation during wheat grain development **A** Motif density of 40 clustered motifs from JASPAR database (Castro-Mondragon et al. 2022) in eight diffACR clusters. **B** Ternary plot showing expression bias of the syntenic genes from three subgenomes. The balanced category: balanced expression of the triad genes. A dominant, B dominant and D dominant: higher expression from A, B or D than the other two orthologs. A suppressed, B suppressed and D suppressed: lower expression from A, B or D than the other two orthologs. **C** Sankey diagram showing expression dynamic of the syntenic genes during grain development. **D** Sankey diagram showing the chromatin accessibility dynamics of the promoter regions (from 2 Kb upstream to 100 bp downstream of TSS) of the syntenic genes during grain development. **E** The subgenome diversified expressions of key transcription factors. **F** Motif density of eight diffACR clusters in each subgenome (* indicate motif density > 1)

Transcription factors (TFs) play important roles in the regulation of gene expression, while ACRs are predominantly TF-binding sites (Liu and Bergmann 2021; Marand et al. 2021). To identify the key TFs responsible for the gene expression dynamics during wheat grain development, we analyzed the enrichments of the 40 JASPAR cluster motifs (Castro-Mondragon et al. 2022) in the eight diffACRs clusters. The results showed that ARF, BPC and ERF were enriched in leaf-specific ACRs; ABI, ATHB, NAC, MYB, SPL, STZ and bZIP were enriched in DAP5s, DAP9s and DAP5&9s; AGL, ARF, E2FA, and REF were enriched in DAP15s, DAP20s and DAP15&20; Dof was enriched in all grain specific ACRs (Fig. 2A), indicating the specific roles of different TF families in different grain developmental stages.

Bread wheat (*Triticum aestivum* L.) consists of A, B and D subgenomes derived from three diploid species, and exhibits improved grain yield and nutritional content relative to diploid and tetraploid wheat (Yang et al. 2014). Transcriptomic expression or dynamic modification asymmetry of wheat chromosomes contributed to wide adaptability of wheat (Li et al. 2019; Ramirez-Gonzalez et al. 2018; Wang et al. 2021a). To study the dynamic patterns of gene expression and promoter accessibilities of homeologs during wheat grain development, we conducted the gene expression bias and promoter accessibility bias analysis by ternary plotting (Fig. 2B). 12,218, 11,802, 11,488, 11,319 and 11,597 of triad genes showed balanced expression, and more than 75%, 79%, 78%, 76%, 81% and 74% triad genes showed balanced promoter accessibilities in leaf, DAP5, DAP9, DAP15 and DAP20, respectively (Supplementary Fig. 4A and B). It should be noted that more unbalanced genes were identified in the growing seeds (DAP5, DAP9, DAP15 and DAP20) than in leaves (Supplemental Data Set3). Among the subgenome differentially expressed genes, the counts of B-suppressed genes and promoters were more than the other groups, indicating B subgenome genes were in weaker positions during these processes. We further found that only 56%–62% genes were balanced in expression among subgenomes in all tissues and few genes were consistent among different stages (Fig. 2C). Similar patterns were also found with promoter accessibilities, which indicate highly diverse regulation among subgenomes during grain development (Fig. 2D). In addition, we conducted a correlation analysis between gene expression and promoter accessibility, which showed the unbalanced genes were positively correlated with differential promoter accessible regions in all samples (Supplementary Fig. 4C).

The diversity of CREs was considered as key reasons of transcriptomic expression differentiations (Buenrostro et al. 2013; Lu et al. 2017). The previous reported key TFs controlling grain development showed dynamic subgenome expression differences in leaf, DAP5, DAP9, DAP15 and DAP20 (Fig. 2E). To identify the specific TF-binding divergence among the three subgenomes, we calculated the densities of TF-binding motifs in different grain specific ACRs among three subgenomes. Although the number of diffACRs in three subgenomes did not show obvious differences (Supplementary Fig. 4D), a large proportion of TF-binding sites showed bias enrichments among the three subgenomes (Fig. 2F). For example, E2FA, CDC, LBD, BPC, ARF and Foxn1 binding sites showed higher enrichment on A subgenome in leaf-specific ACRs. MYB, NAC and TGA binding sites showed higher enrichment on A subgenome in DAP9s and DAP5&9s diffACRs. bZIP, BPC and KUA TF-binding sites showed higher enrichment on A subgenome in DAP15, DAP20 and DAP15&20s groups. ARF, MYB and TGA binding sites were subgenome divergently enriched in DAP15s, DAP20s and AllGs groups. The densities of TF-binding sites were also varied during grain development. For example, NAC, KUA and MYB binding motifs were enriched in A subgenome at the DAP9s, while bias enriched in B and D subgenomes at DAP15s (Fig. 2E). Gene Ontology (GO) enrichment analysis showed that the balanced and unbalanced genes among subgenomes during seed development were enriched in different pathways, the balanced genes were enriched in DNA replication, gene expression and protein folding process, while A suppressed genes were mainly enriched in vitamin biosynthetic process, thiamine biosynthetic process and lipid catabolic process, B-suppressed genes were mainly enriched in carbohydrate homeostasis process, carbohydrate phosphorylation process and RNA modification process, D suppressed genes were mainly enriched in organic substance metabolic process and nitrogen compound biosynthetic process, A dominant genes were mainly enriched in phosphatidylinositol biosynthetic process and carbohydrate catabolic process, B dominant genes were mainly enriched in carbohydrate biosynthetic process and metal ion transport process, D dominant genes were mainly enriched in protein phosphorylation and aminoglycan metabolic process. These enriched GO terms were consistent with the grain development processes (Supplementary Fig. 5). To prove the specific binding of TFs in different tissues, we combined the published genome-wide profiling of TFBSs (transcription factor-binding sites) in common wheat (DAP-seq data) (Zhang et al. 2022) and our ATAC-seq data of different tissues to better identify the potential in vivo TFBSs in different tissues. The results showed that more that 60% TFs’ (NAC6A-1, NAC6B-1, NAC-6D-1 and MYB-7A-3) binding sites were different among various tissues (Supplementary Fig. 6A). In addition, we analyzed the subgenome-divergent regulation by TFs (NAC6A-1, NAC6B-1, NAC-6D-1 and MYB-7A-3), and found that nearly 80% traids showed subgenome-divergent regulations (Supplementary Fig. 6B). These results indicated tissue-specific and subgenome-divergent regulation by multiple TFs during wheat grain development. The high variations of TF-binding sites enrichment bias among subgenomes at different stages suggested the three subgenomes worked coordinately during wheat grain development and may contribute to the improved grain yield and nutritional content after polyploidization.

### Starch and gluten regulatory networks mediated by key TFs

**Fig. 3:**
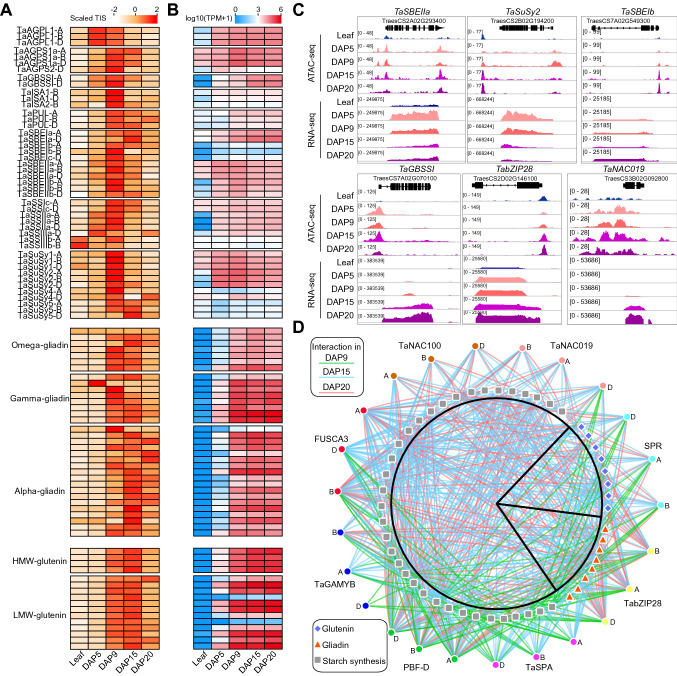
Construction of regulatory networks for starch and gluten biosynthesis by integration the chromatin accessibility and expression data. **A** and **B** Promoter chromatin accessibility dynamics (**A**) and expression dynamics (**B**) of genes involved in glutenin and starch accumulation in leaf and grain samples. Regions from 2 Kb upstream to 100 bp downstream of transcriptional start sites were considered as promoters. **C** Genome browser tracks showing the expression and promoter chromatin accessibility patterns of starch synthesis genes (*TaSBEIIa*, *TaSuSy2*, *TaSBEIb* and *TaGBSSI*) and key TF genes (*TaZIP28* and *TaNAC019*). **D** Regulatory networks involving the starch and gluten biosynthesis genes and key TFs. TF families are shown as circles with different colors. Starch biosynthesis genes are shown as grey square; Gliadin genes as orange triangle; Glutenin genes as blue diamond. TF-to-gene edges are shown with three colors represent three grain phases. DAP9, green; DAP15 blue; DAP20, pink

During the filling stages, expression of genes involved in the starch and gluten biosynthesis pathways were gradually increased. However, the exact *cis*-regulatory elements responsible for the tissue-specific expression were still elusive. We picked up the genes related with gluten protein accumulation and starch biosynthesis regulation including HMW-GS, LMW-GS, alpha-gliadin, gamma-gliadin and *AGPS1*, *AGPL1*, *GBSSI*, *SBEI*, *SBEII*, *SSII*, and *SuSy* in starch biosynthesis (Supplemental Data Set4). We found that the promoter accessibilities of genes involved in starch biosynthesis were higher in seeds than leaves and increased after DAP5, while those of glutenin and gliadin genes were increased after DAP9 (Fig. 3A), which is consistent with the transcriptomic expression patterns (Fig. 3B). Interestingly, the promoter accessibilities of genes in DAP20 were starting decrease while those genes were still highly expressed (Fig. 3A and B). Meanwhile, for some genes highly expressed after DAP9, higher promoter accessibilities could be found at DAP5 (Fig. 3A and B), indicating the changes of promoter accessibilities are prior to the transcriptomic expression changes. Taken together, these results indicated that the chromatin accessibilities were tightly associated with the expression changes of those grain-filling-related genes.

**Fig. 4:**
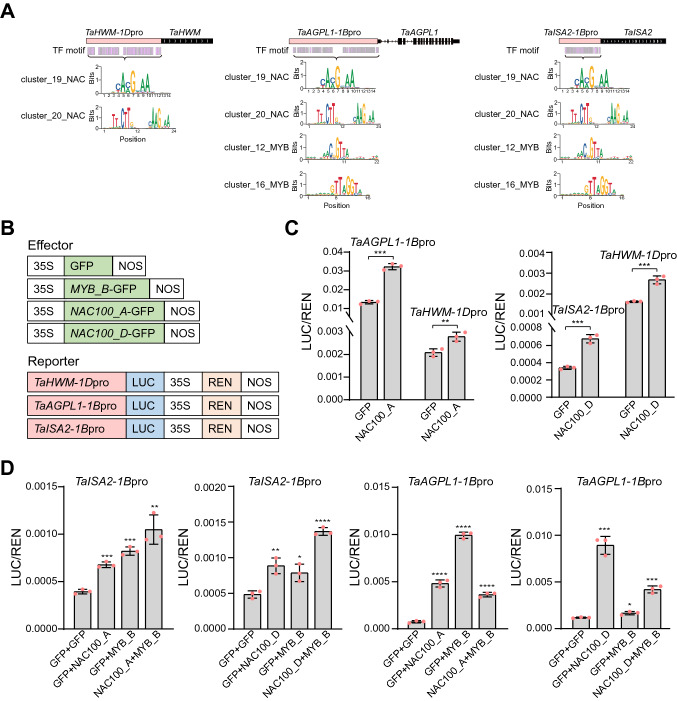
Dual-luciferase transcriptional activity assay to assess the capability of key TFs to transactivate predicted target gene expression. **A** The distribution of key TF-binding motifs in the promoters of starch and protein synthesis genes.** B** Schematic diagrams of the effector and reporter constructs for the dual-luciferase transcriptional activity assay. **C** Reporter assay showed that *NAC100*-*A* or *NAC100*-*D* regulates both starch and protein synthesis pathways. **D** *NAC100* and *MYB-B* coordinately regulate *TaISA2* and *TaAGPL1* genes. LUC/REN indicates the signal ratio of LUC (firefly luciferase) to REN (*Renilla reniformis* luciferase) activity. Data are represented as means ± SD from three replicates. (Student’s *t*-test; *P < 0.05, **P < 0.01)

To find the key TFs controlling grain development, we further calculated the densities of TF-binding motifs in the promoters of genes related with gluten protein accumulation and starch biosynthesis, which suggested that NAC, bZIP and MYB family TFs play important roles (Supplementary Fig. 7). Consistently, the key TFs related with grain development, including TaNAC019, TabZIP28, and TaGAMyb showed increased promoter accessibilities and transcriptomic expression patterns in grains (Fig. 3C and Supplementary Fig. 8). We then constructed a regulatory network based on the chromatin accessibility data and expression pattern of those key TFs and genes involved in starch biosynthesis and protein accumulations. The results showed that the three copies of TaSPA were involved in the networks in DAP9 and DAP15; TaNAC019 and TaNAC100 in DAP15 and DAP20; while the others mostly in all the stages after DAP9 (Fig. 3D). Subgenome specific regulation could be found with FUSCA3, of which the copy from D subgenome were involved in networks in DAP9 and DAP15, while the other two in all the three stages. Similarly, the copy of TaGAMYB from D subgenome were in DAP9 and A subgenome copy in DAP9 and DAP15 while B subgenome copy in all the three stages. The results also revealed most of these key TFs were involved in both starch synthesis and protein accumulations, indicating a complex network between starch and protein synthesis. To further verify the predicted transcriptional regulation network, we conducted the dual-luciferase transcriptional activity assay (Fig. 4A–B and Supplemental Data Set5). The assay showed that the reporter activities driven by the promoters of *TaAGPL1-1B*, *TaHWM*-*1D* and *TaISA2-1B* were markedly activated in the presence of *NAC100*-*A* or *NAC100*-*D*, suggested that *NAC100* regulates both starch and protein synthesis pathways (Fig. 4C). Moreover, *NAC100* and *MYB* could coordinately regulate same targets (*TaAGPL1-1B* and *TaISA2-1B*) (Fig. 4D). In summary, we have explored the chromatin accessibility landscapes and identified the key TFs and regulatory network that were involved in the starch and protein biosynthesis during wheat grain development.

## Discussion

Wheat grain development is critical for the quality and yield of wheat, a series of genes regulated storage protein and starch biosynthesis have been discovered in the previous study (Xiao et al. 2022). Transcriptional regulation of gluten and starch genes involves complex network between *cis-* and *trans-*acting factors, but little is known about this regulation. The accessible chromatin regions often associated with TFs related CREs (Lu et al. 2017; Marand et al. 2021; Pei et al. 2022). Here, we constructed a chromatin accessibility atlas for leaf and four different stages of seed during grain development. This regulatory atlas enabled specific ACR and TF identification at different phase of wheat grain development. We found NAC, bZIP and MYB binding motifs are highly enriched in the grain specific ACRs as previous reported (Gao et al. 2021; Guo et al. 2015; Liu et al. 2020; Song et al. 2020). More importantly, some novel TFs, including REF, Dof and HMG were also discovered highly enriched in the these specific ACRs. Further studies on TFs in those families will help to understand the molecular basis of grain development.

Chromatin accessibilities’ changes are associated with genes’ differential expression. In this study, we combined ATAC-seq with RNA-seq and constructed the dynamic regulatory network during wheat grain development, and further validated the regulatory network by dual-luciferase transcriptional activity assays. We found that the phase-specific ACRs are associated with phase-specific DEGs, and the function of phase-specific genes is related to specific the biological process of specific tissue. The seed-specific genes were highly enriched in carbohydrate and sucrose metabolic process, and seed development processes. We also found the changes of promoter accessibilities of starch and protein biosynthesis genes were prior to their expression changes, indicating those RNAs were likely accumulated rather than rapidly synthesized. Besides, we found the homeolog dynamic transcriptomic expression divergence during wheat grain development (Xiang et al. 2019) are associated with the divergence of promoter accessibilities and the TF-binding sites in the three subgenomes at different phases of wheat grain development. Therefore, those accessible regions in different phases could help to accurately discover the key *cis-* and *trans-*acting factors during wheat grain development. In summary, our data provided a transcriptional regulation resource to understand the complex networks and identified the key regulatory elements and TFs underlying wheat grain development.

## Methods

### Plant material and growth conditions

Wheat (*Triticum aestivum*; BBAADD, 2*n* = 6x = 42) cultivar AK58 was transferred to the greenhouse with conditions day/night cycle set at 16 h (22 °C)/8 h (19 °C) after vernalization for 1 mouth in cold chamber (4 °C). Wheat endosperm was excised by removing the seed embryo at 5 and 9 days after fertilization. Wheat seed was collected at 15 and 20 days after fertilization.

### RNA-seq

The collected wheat endosperm and seed were flash-frozen with liquid N2 immediately. Total RNA was extracted with TRIzol™ Reagent (Invitrogen; 15,596–026) following the manufacturer’s instructions. RNA-Seq libraries were constructed and sequenced by Berry Genomics (Beijing, China), the libraries were sequenced on the Illumina NovaSeq 6000 platform and produced 150-bp paired-end reads.

### ATAC-seq

ATAC-seq was performed as described previously (Lu et al. 2017). For each sample, approximately 1 g flash-frozen wheat samples were chopped with a razor blade in 1 mL ice-prechilled lysis buffer (15 mM Tris–HCl pH 7.5, 20 mM NaCl, 80 mM KCl, 0.5 mM spermine, 5 mM 2-Mercaptoethanol, 0.2% TritonX-100). The chopped slurry containing crude nuclei extract was filtered twice through a 40 μm filter. The crude nuclei were stained with DAPI (sigma, catalog number: D9542) and loaded to a flow cytometer (BD FACSCanto) for selected. Nuclei pellets were obtained after centrifuged and washed with Tris-Mg buffer (10 mM Tris–HCl pH 8.0, 5 mM MgCl_2_). The obtained nuclei were incubated with 3.5 μL Tn5 transposomes in 40 μL TTBL buffer (TruePrep DNA Library Prep Kit V2 for Illumina, Vazyme Biotech co., ltd, TD501) for each sample at 37 °C for 30 min without rotation. We purified the integration products with a NEB Monarch™ DNA Cleanup Kit (T1030S) and then amplified for 10–13 cycles using the NEBNext Ultra II Q5 master mix (M0544L). PCR cycles were determined as described previously (Lu et al. 2017). Amplified libraries were purified with Hieff NGS® DNA Selection Beads (Yeasen, 12601ES03) to remove free index primers.

### Processing of RNA-seq data

Raw reads were preprocessed by fastp for filtering low-quality reads and adapter trimming. The remaining reads were aligned to the wheat reference genome using hisat2 with default parameters. All aligned reads were sorted by SAMtools v1.3.1 (Swiezewski et al. 2009). To compare the expression from different phases, TPM was calculated by TPMCalculator. Differential expressed genes (DEGs) were defined as adjusted *P*-value < 0.01 and log2(FoldChange) > 1, which determined by DESeq2.

### Processing of ATAC-seq data

Raw reads were trimmed with fastp with default parameters. Trimmed reads were aligned to the wheat reference genome using Bowtie v2.2.4 with the following parameters: “bowtie2-X 1000 –very-sensitive”. Aligned reads were sorted using SAMtools v1.3.1 (Swiezewski et al. 2009) and clonal duplicates were removed using Picard version v2.16.0 http://broadinstitute.github.io/picard/). Peak calling was performed as described previously. A black list was first generated using the control samples as input with MACS2. The raw peaks were first called with MACS2 with the following parameter “--keep-dup all --nomodel --extsizes 150 --shift -75” (Zhang et al. 2008); then split into 150 bp bins with 50 bp overlapping; bins passed filtering by a Tn5 integration site density cutoff were selected and merged with bedtools with “– *d* 150”; peaks overlapped with the blacklist peaks, or homologous to plant organelles DNA (NCBI) were discard and the rest were considered as high-quality ACRs. Bigwig files were used to visualize the peaks in the integrative genomics viewer.

### Dual-luciferase reporter gene assay

Dual-luciferase reporter gene assay was performed in *N. benthamiana* leaves by *Agrobacterium* tumefaciens-mediated transient expression system. The promoters of *TaAGPL1*-*1B*, *TaHWM*-*1D* and *TaISA2*-*1B* were amplified from AK58 genomic DNA and inserted into the pGreenII 0800-LUC vector as reporter plasmids (Hellens et al. 2005). The CDS of *NAC100-A*, *NAC100-D* and* MYB-B* were cloned in frame into the pEG1300-GFP vector as effector plasmids. *N*. *benthamiana* plants at the six-leaf stage were co-infiltrated with *A. tumefaciens* strain GV3101 harboring different combinations of these plasmids. The dual-luciferase reporter assay system (Yeasen, China) was used to measure LUC and REN activities and three independent replications were conducted.

## Supplementary Information

Below is the link to the electronic supplementary material.

Supplementary file1 (PDF 3753 KB)

Supplementary file2 The lists of eight diffACR groups (XLSX 3244 KB)

Supplementary file3 The lists of eight DEG groups (XLSX 455 KB)

Supplementary file4 List of the unbalanced expressed genes among subgenomes during seed development (XLSX 16 KB)

Supplementary file5 List of key TFs and wheat grain related genes (XLSX 16 KB)

Supplementary file6 List of primers used in this study (XLSX 10 KB)

## Data Availability

All the raw sequencing data generated during the current study are available in the Gene Expression Omnibus (GEO) database (https://www.ncbi.nlm.nih.gov/geo) under accession number GSE214739.
