# Supplementary material for: Chromatin accessibility landscapes revealed the subgenome-divergent regulation networks during wheat grain development
Source: aBIOTECH. 2023 Feb 10;4(1):8–19. doi: 10.1007/s42994-023-00095-8 (PMC10199822; doi:10.1007/s42994-023-00095-8)
Supplement: Supplementary file 1 — Supplementary file1 (PDF 3753 KB) [file 42994_2023_95_MOESM1_ESM.pdf]

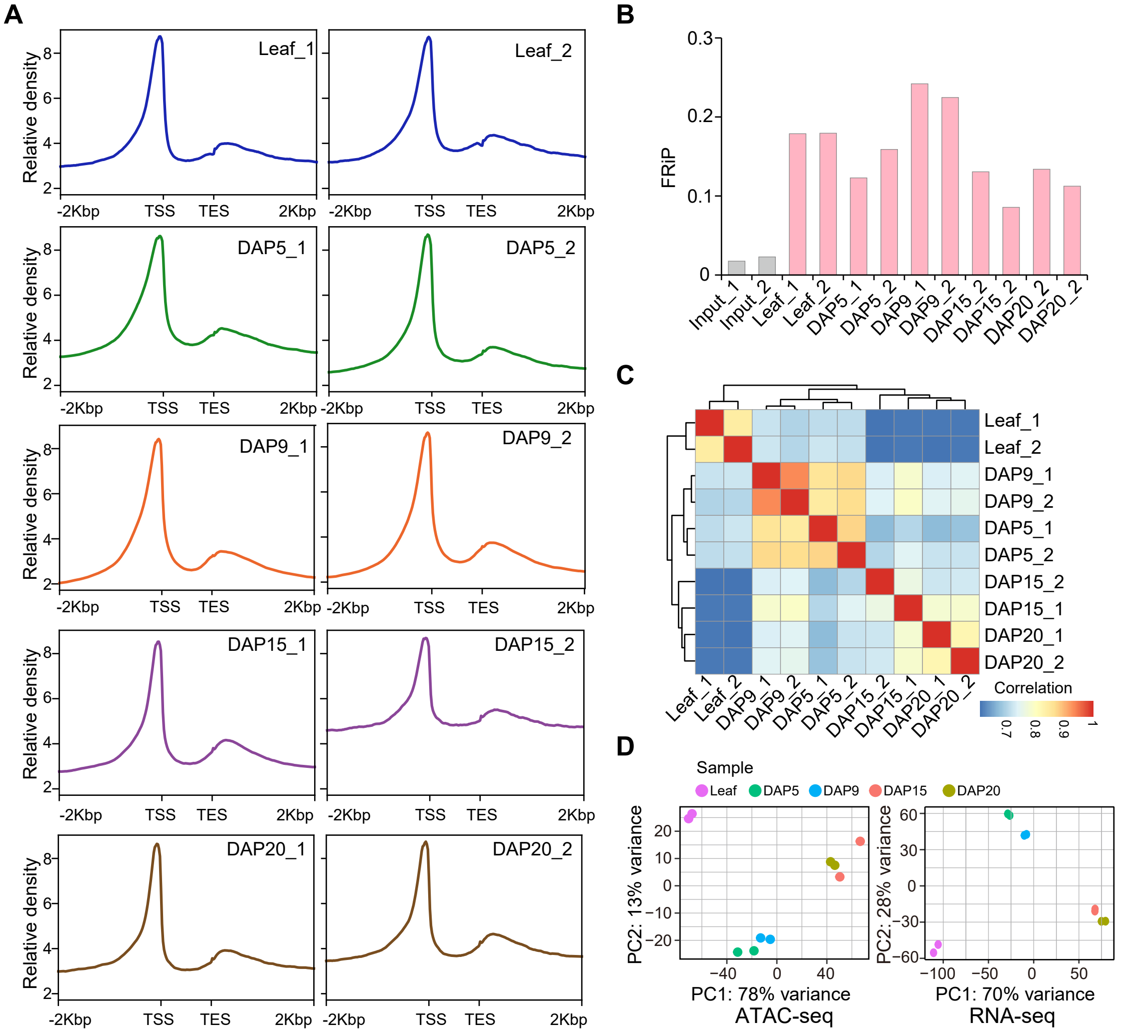

Supplemental Fig. 1 Quality control of ATAC-seq and RNA-seq samples.

A Distribution of ATAC-seq reads around Transcriptional Start sites (TSSs) and Transcriptional End Sites (TESs) in the five wheat samples. **B** Fraction of Reads in Peaks (FRiP) for all ATAC-seq data used in this study. **C** Heatmap showing the correlation among all ATAC-seq samples. **D** PCA analysis of ATAC-seq and RNA-seq data.

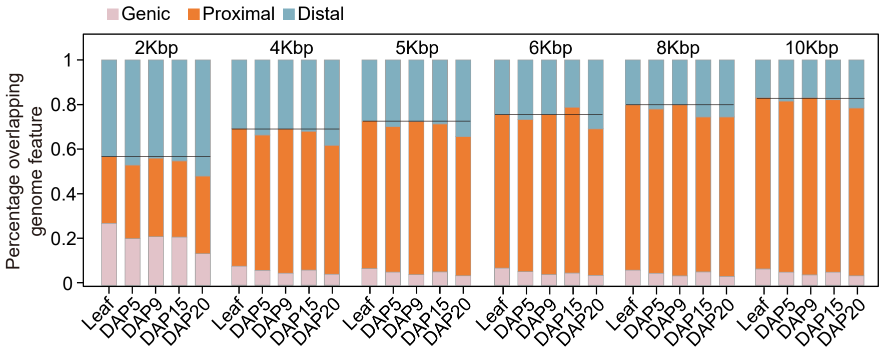

Supplemental Fig. 2 Distribution of ACRs on different genomic features with different cutoffs. Genic, ACRs with their centers in gene bodies; The proportion of ACRs located in proximal and distal were categorized with different cutoffs. Proximal, ACRs with their centers <= n Kbp upstream of TSS or <= n Kbp downstream of TES; Distal, ACR with their centers > n Kbp away from genes.

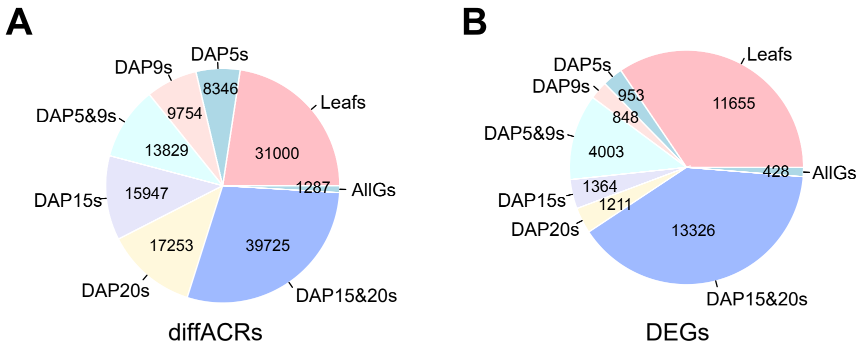

Supplemental Fig. 3 The number of diffACRs (A) and DEGs (B) in each cluster.

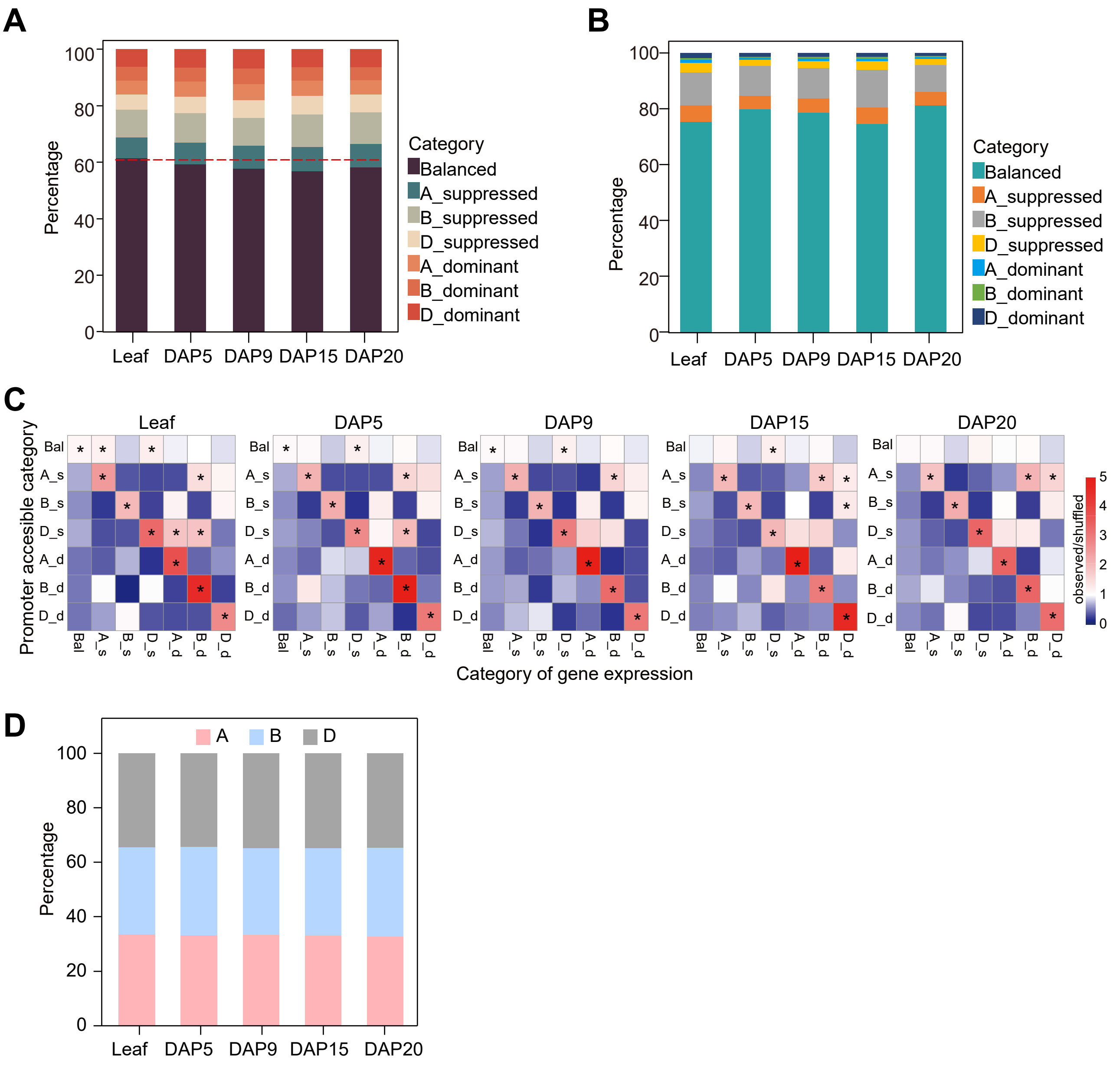

Supplemental Fig. 4 Subgenome-divergence of genes' expression levels and promoter accessibilities.

A The percentage of balanced and unbalanced expressed triad genes according to Fig 2B. **B** The percentage of different promoter accessibility type of triad genes. **C** Correlation analysis of unbalanced expressed genes and differential promoter accessible regions. Bal: Balanced, A_s: A suppressed, B_s: B suppressed, D_s: D suppressed, A_d: A dominant, B_d: B dominant, D_d: D dominant. **D** The distribution of diff ACRs on the three subgenomes.

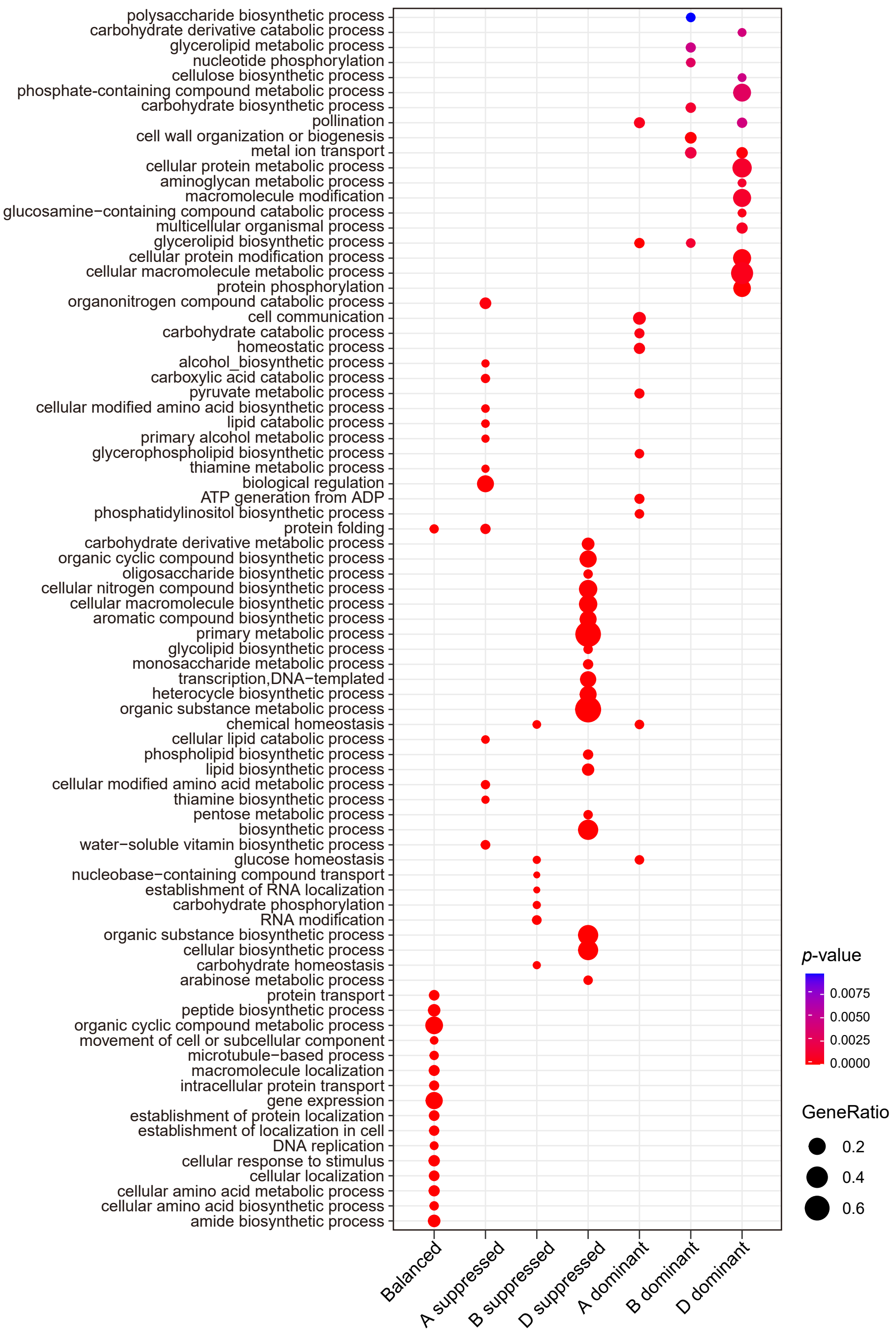

Supplemental Fig. 5 Gene ontology (GO) enrichment analysis for the balanced and unbalanced expressed genes during seed development.

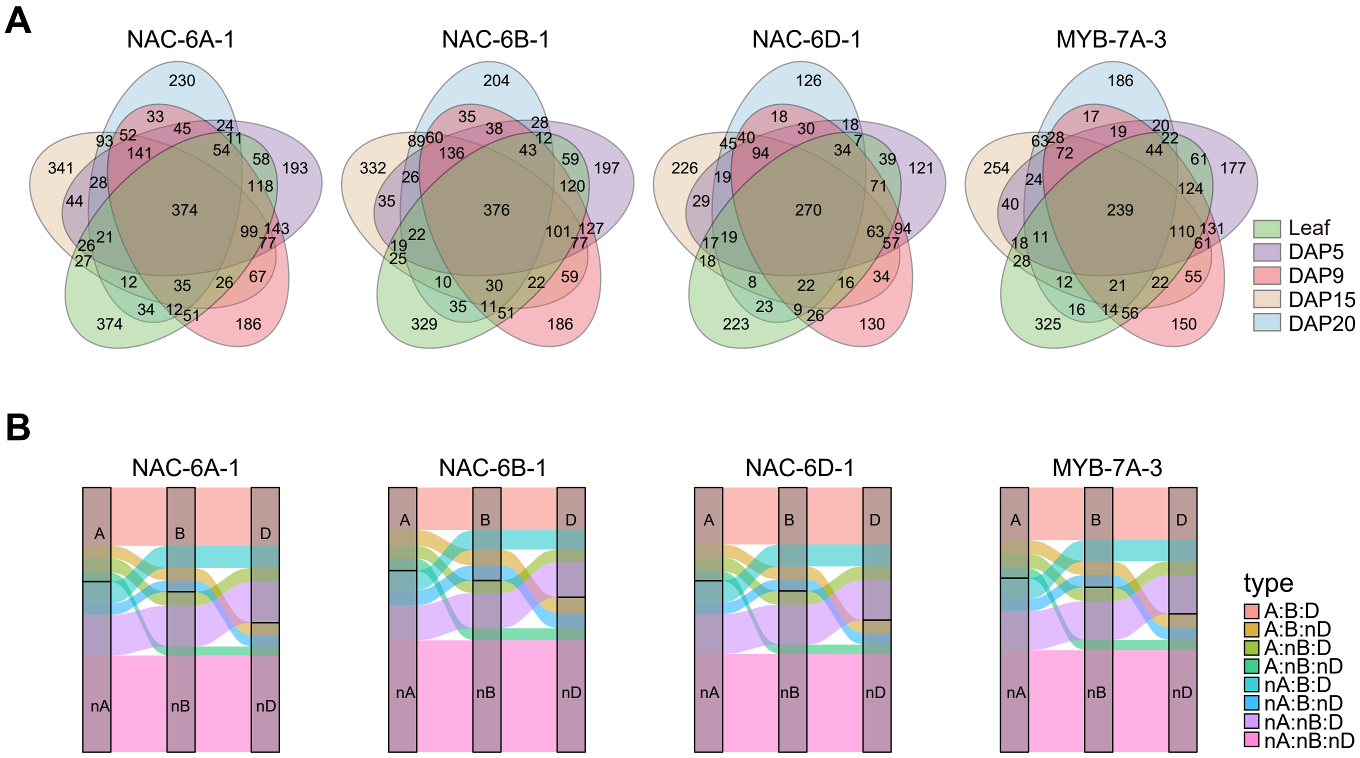

Supplemental Fig. 6 Tissues specific and subgenome divergent regulation by TFs during wheat grain development.

A Specific binding of TFs to different tissues. B Subgenome divergent regulation by TFs. A, B, D: TF binding on the subgenome. nA, nB, nD: TF not binding on the subgenome.

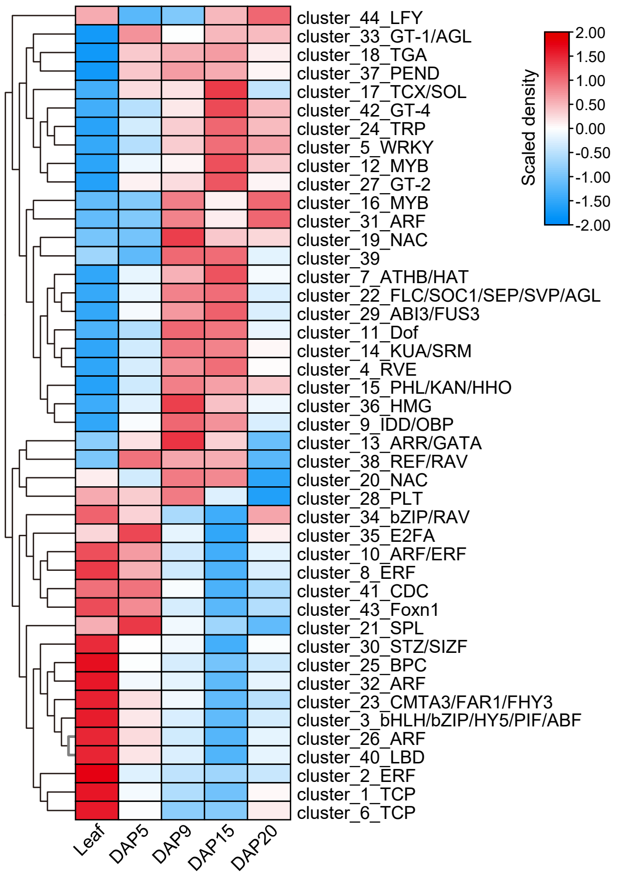

Supplemental Fig. 7 Motif density of ACRs in the promoters of genes related with gluten accumulation and starch biosynthesis.

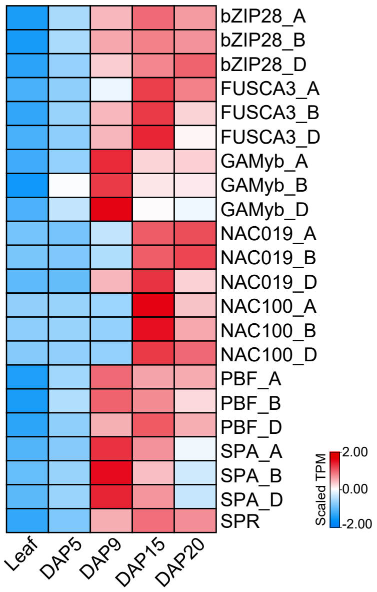

Supplemental Fig. 8 Heatmap displays the expression pattern of key TFs.
